# Emotional and Phenomenological Properties of Odor-Evoked Autobiographical Memories in Alzheimer’s Disease

**DOI:** 10.3390/brainsci9060135

**Published:** 2019-06-10

**Authors:** Ophélie Glachet, Mohamad El Haj

**Affiliations:** 1 University of Lille, CNRS, CHU Lille, UMR 9193 SCALab-Sciences Cognitives et Sciences Affectives, F-59000 Lille, France; 2 Laboratoire de Psychologie des Pays de la Loire (EA 4638), Université de Nantes, F-44000 Nantes, France; mohamad.elhaj@univ-nantes.fr; 3 Unité de Gériatrie, Centre Hospitalier de Tourcoing, F-59200 Tourcoing, France; 4 Institut Universitaire de France, F-75000 Paris, France

**Keywords:** Alzheimer’s disease, autobiographical memory, depression, emotion, olfaction, subjective reliving

## Abstract

Autobiographical memory, which contains all personal memories relative to our identity, has been found to be impaired in Alzheimer’ Disease (AD). Recent research has demonstrated that odor may serve as a powerful cue for the recovery of autobiographical memories in AD. Building on this research, we investigated emotional characteristics (arousal and valence) and subjective reliving of odor-evoked autobiographical memories in AD. We also investigated the relationship between these characteristics and depression. To this end, we invited participants with mild AD and controls to retrieve autobiographical memories after odor exposure or without odor. Results showed higher arousal, subjective reliving and more positive memories after odor exposure compared with the odor-free condition, these differences being observed only in AD participants. We also found that emotion (arousal and valence) and subjective reliving triggered by odor were associated with depressive symptoms in AD. These findings demonstrate that odor may be a useful cue to trigger more detailed, vivid and positive events in AD.

## 1. Introduction

Autobiographical memory is an essential part of the human memory system as it allows the construction and maintenance of self-awareness, personal knowledge and self-image [1], and can be defined as the ability to relive past personal events. Autobiographical memory has been found to be impaired in Alzheimer’s disease (AD) [2,3,4,5,6]. Autobiographical memory compromise in AD is characterized by an overgenerality, i.e., a reduced ability of AD patients to produce specific memories [4,7,8,9,10,11,12,13,14]. Decline of autobiographical memory in AD is also associated with changes in the strength and the quality of the sense of identity in patients [15]. As a result of autobiographical memory impairment, AD patients demonstrate difficulty in mentally reliving past events with the perceptual, sensory and conceptual details of the original event, replaced by a general feeling of knowing or familiarity [2,16,17,18,19,20]. 

Studies have tried to alleviate impairment of autobiographical memory in AD by focusing on sensory cues. Some studies have shown that powerful perceptive cues such as music and odor have a beneficial impact on the involuntary retrieval of autobiographical memories in AD, principally by diminishing the time taken to retrieve these memories [2,8,21]. Involuntary autobiographical memories were described by [22] as conscious memories of personal events that come to mind spontaneously. These authors suggest that involuntary retrieval may promote a direct link between the cue and the memory trace, thus avoiding the complex recovery process involved in voluntary autobiographical remembering [1,8,23,24]. Involuntary retrieval may be triggered by odor and, unlike other modalities, olfactory stimuli are not relayed by the thalamus during cortical processing [25]. Olfactory signals are directly connected with two key structures involved in memory and emotions: the amygdala and the hippocampus [26], which may explain why odor-evoked autobiographical memories are typically emotional and vivid in general populations [27]. 

Odor has been found to be unique in its ability to enhance the quality of autobiographical memory. Research has suggested that odor exposure has a beneficial effect on the recovery of phenomenological details associated with the evocation of autobiographical memories. Odor-evoked autobiographical memories are characterized by significant mental time travel [28,29,30], i.e., the feeling of being brought back to the past at the moment of the original event [31]. Odor-evoked autobiographical memories are also more vivid than memories cued by other sensory modalities [32,33,34]. Interestingly, neuroimaging studies have shown that odor-evoked memories are characterized not only by the activation of brain areas involved in memory recovery and cortical processing of olfactory stimuli, but also by the recruitment of brain regions usually activated during visual imagery (olfactory gyrus and precuneus) and emotions (limbic and tempopolar regions) [32,35], which may explain why involuntary memories are described as more evocative. 

Besides being evocative, odor-evoked autobiographical memories trigger significant emotional content [28,36,37,38]. Herz and Schooler [29] found that odor-evoked autobiographical memories were rated as more emotional than those evoked by verbal or visual cues. Regarding emotional valence, research has shown that odor-evoked autobiographical memories are more “pleasant” than those elicited by other modalities in young healthy adults [32] and older healthy adults [39]. The power of odor in eliciting positive memories could be due to the neuronal processing of olfactory memories. Arshamian et al. [32] reported that odor-evoked autobiographical memories were associated with an increased cortical activation in the temporal gyrus and the temporal pole. The temporal pole is known to be involved in the processing of pleasant memories. Arshamian et al. [32] suggested that increased activity in the temporal lobe underlies the positivity of odor-evoked autobiographical memories. 

To our knowledge, two studies have already demonstrated the beneficial effects of odor on autobiographical remembering in AD. In a recent study, El Haj et al. [2] investigated the involuntary nature of autobiographical memory in AD triggered by music and odor. AD patients showed improved specificity, emotional experience, retrieval time and mental time travel when memories were cued by odor compared to an odor-free condition. The authors observed shorter retrieval time for memories cued by odor than for those evoked by no cue. This study was the first to demonstrate the beneficial effect of olfactory cueing in AD and has important clinical implications, as olfactory cues could serve as a useful tool to stimulate autobiographical memory in this pathology. In a related vein, Glachet et al. [21] found similar results for specificity and recovery time. They also showed that compared with memories evoked without odor, odor-evoked autobiographical memories were characterized by a higher subjective reliving in AD. Interestingly, there was no significant effect of odor in terms of specificity and reminiscence in heathy older adults, suggesting that this type of cueing is particularly efficient when autobiographical memory is impaired. While these studies demonstrated positive effects of odor on autobiographical in AD, they did not evaluate the impact of olfactory cueing on arousal and emotional valence of these memories. Therefore, the main aim of the present study was to assess whether odor exposure may enhance the subjective reliving and the emotional properties of autobiographical memories in AD. 

In our view, it would be of interest to evaluate whether odor may help patients with AD to retrieve positive autobiographical memories, despite the prevalence of depression in AD. Generally speaking, AD is associated with several behavioral and neuropsychiatric symptoms such as apathy and depression [40,41], and the latter affects at least 50% of AD patients [42]. Critically, research has demonstrated that patients with emotional disorders such as depression tend to retrieve overgeneral memories with little access to specific details [43,44]. Moreover, Young et al. [45] demonstrated reduced activity in the left amygdala during the retrieval of positive autobiographical memories in patients with depression and considered that hypoactivity in the left amygdala might be a marker of depression. We therefore evaluated the relationship between depression and phenomenological characteristics (e.g., emotional valence) of odor-evoked autobiographical memories in AD. 

To summarize, while previous research has demonstrated positive effects of odor on autobiographical in AD [2,21], it did not evaluate the impact of olfactory cueing on arousal and emotional valence of these memories. We therefore evaluated whether odor exposure may enhance the subjective reliving and the emotional properties of autobiographical memories in AD. We also evaluated the relationship between depression and phenomenological characteristics (emotional valence, arousal, subjective reliving and specificity) of odor-evoked autobiographical memories in AD. We expected that odor-evoked autobiographical memories would be more positive and be accompanied by more arousal and subjective reliving than memories evoked without odor in AD. Given the prevalence of depression in AD patients [42] and its critical impact on autobiographical memory [43,44], we also expected a negative correlation between depression and the phenomenological characteristics of autobiographical memories (arousal, emotional valence subjective reliving and specificity) in this group. To this end, we invited AD and control participants to retrieve autobiographical memories in an odor compared to an odor-free condition, and then evaluated them in terms of emotion (arousal and valence), subjective reliving and specificity.

## 2. Methods

### 2.1. Participants 

The study included 25 participants at the mild stage of AD and 23 healthy elderly adults. The sample size is similar to previous studies [2,21,46] using a close experimental design, and was sufficient to observe significant differences between AD and control participants in both experimental conditions (odor and odor-free). From the original sample of 36 AD participants, six participants were excluded from the study due to olfactory impairment, seven due to the very low scores on the cognitive battery, and one due to inability to follow the instructions provided. 

The AD participants were recruited from local retirement homes and were diagnosed with probable AD dementia by a neurologist or a geriatrician, based on the National Institute on Aging-Alzheimer’s Association criteria [47]. Control participants were often spouses or relatives of AD patients. All participants were French native speakers and reported no visual or auditory impairment. As shown in Table 1, both groups were matched according to age and education level. AD participants with dementia in which memory disorders were not in the foreground (i.e., mixed dementia or fronto-temporal dementia) were ineligible. For all participants, exclusion criteria were other neuropsychological or psychiatric illness, or a history of alcohol and drug use. Demographic characteristics, neuropsychological and clinical assessments are presented in Table 1. All participants provided written informed consent. They were free to participate in the study and could withdraw whenever they wished. 

### 2.2. Cognitive and Clinical Assessment 

In a preliminary session, we evaluated general cognitive efficiency, episodic memory and depression. All scores are shown in Table 1. 

General cognitive functioning was assessed with the Mini-Mental State Examination (MMSE) [48], with a maximum score of 30 points. The Grober and Buschke task [49] was used to evaluate episodic memory. In this task, participants were asked to learn and retain 16 words, each belonging to a different semantic category. After an immediate recall, there was a 20-second distraction phase during which they had to count backwards from 374. The distraction phase was followed by a free recall of the 16 words for two minutes. Episodic memory performance was evaluated as the number of words /16 properly recalled during the free recall. Finally, we assessed depression with the Hospital Anxiety and Depression Scale (HAD) [50] to determine whether the potential presence of depressive affects could impact the emotional valence of autobiographical memory. The HAD consists of seven items scored on a four-point Likert scale from 0 (not present) to 3 (considerable). The maximum score was 21 points and the cut-off for definite depression was set at > 10/21 points. 

### 2.3. Experimental Procedure

Participants were tested individually in two sessions: without odor and after odor-exposure, separated one week apart. The order of the two conditions was counterbalanced for all participants. In the odor-exposure condition, participants were presented with a bottle of scented oil. They were asked to move the bottle under their nose, and then to breathe normally through the nose. Since one of the main clinical signs in AD is the loss of olfactory abilities [51,52,53], we used cinnamon as it was already found not to be lost or declined in AD patients compared to control participants [54]. Directly after odor exposure, participants were asked to retrieve an autobiographical memory with the following instruction: “Please think about an event in your life that has special meaning for you. It should not have lasted more than 24 hours and should be as detailed as possible: Where and when did it happen? What did you do? Who was present? What were your feelings?”. The participants were allowed three minutes to describe their memories. This time limit was adopted to avoid certain biases such as redundancy and distractibility. In the control condition, the autobiographical instruction was the same, but no odor was presented. After the memory recall, participants were invited to score their memories on the subjective experience and emotion scales described in the following sections. 

### 2.4. Evaluation of Subjective Reliving 

After autobiographical retrieval, participants were asked to rate their subjective reliving on a scale based on the one used by [55], and adapted in French. The subjective reliving scale was composed of six subcomponents, each comprising two items. After memory retrieval, participants rated mental time travel (“I feel like I travelled back to the time it happened”; “I feel like I’m reliving this moment as it happened), truthfulness of memory (“I can totally remember it and not only know that it happened”; “I think it happened as I remember”), imagery (“I can see it in my mind”; “I can hear it in my mind”), emotion (“I feel the emotions now as they were at the time”; “I feel like my mood has changed”), identity (“It is an important memory in my life”; “Since it happened, I have been thinking about the event or talking about it”), and spatio-temporal specificity (“I can remember where it happened”; I can remember when it happened”). Participants were asked to rate each item on a 4-point Likert scale (1—not at all true; 2—slightly true; 3—moderately true; 4—absolutely true), according to their memories. The maximum score on the subjective reliving scale was 48 points. 

### 2.5. Evaluation of Emotion

The emotional aspects of autobiographical memories were scored by the participants after the memory recall. We used the Self-Assessment Manikin (SAM) [56], allowing for a subjective evaluation of both arousal and emotional valence. Participants were asked to rate these two dimensions of their memories compared to pictorial representations on a nine-point Likert scale. Considering emotional valence, participants were invited to use the extremely happy SAM rating if the memory involved a very positive content, the extremely unhappy SAM rating if the content of the memory was very negative, and to use the intermediate SAM ratings for memories including intermediate positive or negative emotions. For arousal, they were instructed to use the very calm SAM rating if the content of the memory included no arousal, the extremely excited representation if the memory included extreme emotions, and to use intermediate SAM ratings for memories including intermediate levels of arousal. The maximum score for arousal and emotional valence was 9 for each. 

### 2.6. Evaluation of Specificity 

Autobiographical specificity was measured by the experimenter from the narrative of the participants. The performance was scored on the TEMPau scale (Test Episodic de Mémoire du Passé) [57], an instrument based on classic autobiographical memory evaluation [58] that is widely used as a reliable measure of autobiographical memory in AD [2,3,8,21,59,60,61,62], and adapted in French. For each memory, we attributed zero if there was no memory or only general information about a theme (i.e., I was an adolescent). One point was attributed for an extended event without spatio-temporal context (i.e., I was training every week); two points for an extended event situated in time and space (i.e., I was training every Monday and Thursday at the local stadium); three points for a specific event lasting less than 24 hours, and situated in time and space (i.e., I was first in the race organized by my school); and four points for a specific memory with phenomenological details such as feelings, thoughts, visual imagery and emotion (i.e., I was very happy). The maximum score for each memory was 4 points. Autobiographical memory specificity was also categorized by an independent rater who was not blind to the hypothesis. Using Cohen’s Kappa (k) [63], inter-rater agreement coefficient was high (k > 0.86). Disagreements were discussed until a consensus was reached.

### 2.7. Statistical Analysis

We compared autobiographical characteristics (i.e., subjective reliving, arousal, emotional valence and specificity) between AD and control participants for each condition (i.e., odor exposure condition and odor-free condition). We also compared these characteristics between the odor exposure and odor-free conditions for each population. We used non-parametric tests due to the non-normal distribution of data, as shown by the Kolmogorov–Smirnov test. We first compared the differences between AD and control participants in the odor and in the odor-free condition using the Mann–Whitney *U* test. We then performed a within group comparison between both condition in AD and in control using the Wilcoxon signed-rank test. Results are reported with effect size: Cohen’s *d* = 0.2 refers to a small effect size, Cohen’s *d* = 0.5 a medium effect size and Cohen’s *d* = 0.8 a large effect size [64]. Effect size was calculated for non-parametric tests according to the recommendations of [65], and [66]. To evaluate whether odor exposure triggered more positive than negative or neutral autobiographical memories, we used a cut-off score on the SAM valence scale (5 points: neutral memories; <5: negative memories; >5: positive memories). According to the cut-off score, we counted the number of positive, negative and neutral autobiographical memories in the odor and the odor-free conditions for all participants.

To investigate our last hypothesis, we conducted a correlation between depression scores and autobiographical memory characteristics (arousal, emotional valence, subjective experience and specificity). For all analyses, significance level was set at *p* < 0.05. 

## 3. Results

### 3.1. Poor Autobiographical Retrieval in AD

**Figure 1:**
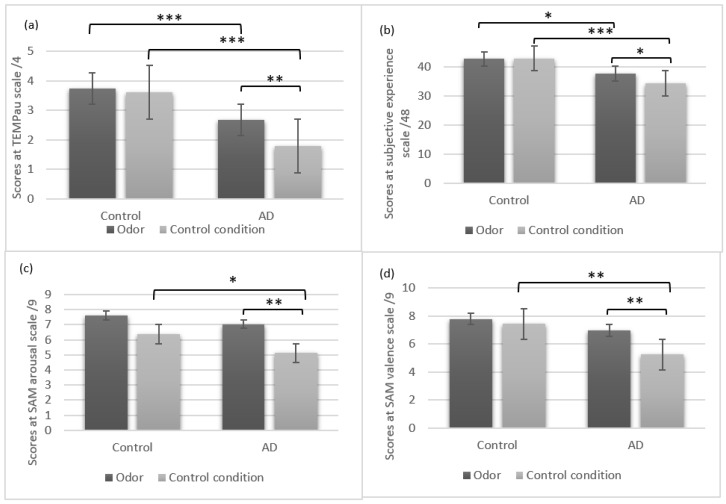
Means and standard errors in AD and control participants for (**a**) specificity, (**b**) subjective reliving, (**c**) arousal and (**d**) emotional valence in the odor and the odor-free conditions. Differences between group and condition were significant at: * *p* < 0.05, ** *p* < 0.01, *** *p* < 0.001.

Compared with controls, AD participants showed less specificity in the odor-free condition (*Z* = −4.98, *p* < 0.001, *Cohen’s d =* 2.21), and after odor exposure (*Z* = −3.69, *p* < 0.001, *Cohen’s d* = 1.20), (Figure 1a). AD participants also showed less subjective reliving than controls in the odor-free condition (*Z* = −3.39, *p* < 0.001, *Cohen’s d* = 1.31) and in the odor condition (*Z* = −2.49, *p* < 0.05, *Cohen’s d* = 0.81), (Figure 1b). As shown in Figure 1c, control participants reported more arousal than AD participants in the odor-free condition (*Z* = −2.09, *p* < 0.05, *Cohen’s d* = 0.54). However, no significant difference was observed in the odor condition (*Z* = −0.71, *p* > 0.05, *Cohen’s d =* 0.28). Similarly, control participants rated their memories as more positive than AD in the odor-free condition (*Z* = −3.14, *p* < 0.01, *Cohen’s d* = 1.06). However, no significant difference was observed in the odor condition (*Z* = −1.55, *p* > 0.05, *Cohen’s d =* 0.44), (Figure 1d).

### 3.2. Odor Exposure is Associated with High Arousal and Positive Emotional Valence in AD

In AD participants, analysis showed higher arousal after odor exposure than in the odor-free condition (*Z* = −3.18, *p* < 0.01, *Cohen’s d =* 0.75). However, no significant differences were observed in control participants (*Z* = −1.89, *p* > 0.05, *Cohen’s d =* 0.49). Similarly, AD participants rated their memories as more positive after exposure to odor than in the odor-free condition (*Z* = −2.75, *p* < 0.01, *Cohen’s d =* 0.65). However, no significant differences were observed in control participants (*Z* = −0.74, *p* > 0.05, *Cohen’s d =* 0.17).

A two-groups (AD and control) by two conditions (odor and odor-free) mixed analysis of variance ANOVA was constructed on arousal and emotional valence scores. Regarding the arousal scores, results showed a significant group effect *F*(1,46)= 4.16, *p* = 0.04, with control participants eliciting more emotional memories than AD participants. The condition effect was also significant *F*(1,46) = 12.09, *p* < 0.001, participants producing more emotional memories in the odor than in the odor-free condition. No significant interaction effect between group and condition was found *F*(1,46) = 0.61, *p* > 0.1. Results also showed a significant group effect for the emotional valence *F*(1,46) = 13.45, *p* < 0.001, with control participants reported more positive autobiographical memories than AD participants. The condition effect was also significant *F*(1,46) = 6.31, *p* = 0.01, participants producing more positive autobiographical memories in the odor compared to the odor-free condition. However, no significant interaction effect between group and condition was found for emotional valence *F*(1,46) = 2.28, *p* = 0.1. 

Analysis also demonstrated a higher proportion of positive autobiographical memories (*χ*² (1) = 3.27, *p* < 0.05; Cramer’s *V* = 0.36) and a lower proportion of negative autobiographical memories (*χ*² (1) = 5.14, *p* < 0.05; Cramer’s *V*= 0.45) in AD after odor-exposure compared to the odor-free condition (Table 2). We found no significant difference in the proportion of positive autobiographical memories (*χ*² (1) = 0, *p* > 0.05; Cramer’s *V* = 0), nor in the proportion of negative autobiographical memories (*χ*² (1) = 0.5, *p* > 0.05; Cramer’s *V* = 0.14) between the two conditions in control participants. 

### 3.3. Odor Exposure is Associated with High Autobiographical Specificity and Subjective Reliving in AD 

Analysis showed higher specificity in AD participants after odor exposure than in the control condition (*Z* = −2.97, *p* < 0.01, *Cohen’s d =* 0.70). However, no significant differences were observed in control participants (*Z* = −0.83, *p* > 0.05, *Cohen’s d =* 0.17). AD participants also showed an improved subjective experience after odor exposure than after the control condition (*Z* = −2.38, *p* < 0.05, *Cohen’s d =* 0.48). No difference was found for subjective experience between odor and odor-free conditions in the control participants (*Z* = −0.05, *p* > 0.05, *Cohen’s d =* 0.04). 

A two group (AD and control) by two conditions (odor and odor-free) mixed analysis of variance ANOVA was conducted for autobiographical specificity and subjective reliving. Regarding the specificity scores, we found a significant group effect *F*(1,46) = 59.56, *p* < 0.001, with control participants produced more specific autobiographical memories than AD participants. The condition effect was also significant *F*(1,46) = 8.15, *p* = < 0.01, participants reported more specific memories in the odor compared to the odor-free condition. A significant interaction effect between group and condition was found for specificity *F*(1,46) = 4.18, *p* < 0.05. Regarding the subjective reliving, analysis demonstrated a significant group effect *F*(1,46) = 22.21, *p* < 0.001, with control participants evoked more vivid memories than AD participants. We found no condition effect for subjective reliving *F*(1,46) = 1.2, *p* > 0.05, and no significant interaction was found between group and condition *F*(1,46) = 1.55, *p* > 0.05. 

### 3.4. Relationship between Depression and Autobiographical Memory Characteristics after odOr Exposure in AD

Depression scores in AD patients were negatively correlated with emotional valence after odor exposure (*r* = −0.44, *p* < 0.05) but not in the odor-free condition (*r* = 0.11, *p* > 0.05). The negative correlation meant that the higher the depression score was, the more autobiographical memories were described as negative. In AD participants, depression scores were negatively correlated with subjective reliving (*r* = −0.48, *p* < 0.05) and arousal (*r* = −0.44, *p* < 0.05) after exposure to odor, but not in the odor-free condition, with respectively *r* = 0.16, *p* > 0.05 and *r* = −0.28, *p* > 0.05. We found no significant correlation between depression score and specificity in AD participants, either in the odor (*r* = 0.03, *p* > 0.05) or in the odor-free condition (*r* = 0.07, *p* > 0.05).

## 4. Discussion

The main aim of this study was to investigate the emotional qualities and the subjective reliving associated with odor-evoked autobiographical memories in AD. We also investigated the relationship between depression and the characteristics of autobiographical retrieval (emotion, subjective reliving, and specificity) in AD. To this end, AD and control participants were invited to retrieve autobiographical memories after odor exposure or in an odor-free condition. We found higher arousal, subjective reliving and specificity as well as more positive evocations for odor-evoked autobiographical memories than for memories evoked without odor. These differences were observed in AD participants but not in controls, suggesting that odor exposure is a particularly useful tool to improve the quality of autobiographical retrieval in AD. Interestingly, odor exposure improved the number of positive memories and decreased the number of negative memories compared to the control condition in AD participants but not in controls. Finally, we observed a negative correlation between depression scores and several characteristics of odor-evoked autobiographical memories (emotional valence, arousal and subjective reliving) in AD patients, thus demonstrating an association between depression and the phenomenological qualities of odor-evoked autobiographical memories in AD. 

Several studies suggest that odor could serve as a powerful cue to relive past events by increasing the quality of autobiographical memory. For instance, odor exposure was found to improve the amount of phenomenological details as well as the emotional experience associated with memories in healthy adults [28,29,32,33,38,39]. The present study replicates previous findings in AD by demonstrating a beneficial effect of odor on specificity and subjective reliving [2,21]. 

Our study is the first to demonstrate the effects of odor on both arousal and emotional valence. To our knowledge, no published study has compared the impact of odor exposure on these two emotional components in AD. Previous studies have focused on the “pleasantness” of odor-evoked autobiographical memories in healthy populations [29,32,39,67]. However, they did not investigate the proportion of positive and negative autobiographical memories indexed by an odor. To investigate the impact of olfactory stimulation on the proportion of positive and negative autobiographical memories, we applied a cut-off score on the emotional valence scale. Interestingly, the number of positive memories was higher and the number of negative memories was lower after odor exposure in AD participants but not in controls. These results have an important clinical implication because this type of memory may help AD patients to focus on the most positive events in life. Odor-evoked autobiographical memories might therefore help them to maintain a positive self-image. 

Another important finding of our study was that odor-evoked autobiographical memories were associated with more arousal in AD, compared to memories evoked without odor. Several studies have attributed the effect of odor on autobiographical memory retrieval to the link between arousal and information associated with the affective reaction [67,68,69]. Therefore, it is likely that the increased arousal triggered by odor-evoked autobiographical memories helps AD patients to retrieve more phenomenological details compared to an odor-free condition. 

Another characteristic of odor-evoked autobiographical memories is their subjective reliving, as they typically trigger significant mental time travel [28,29,30] and are described as particularly vivid [32,33,34]. However, subjective experience is a multidimensional construct involving several components that are selectively impacted by odor exposure. For instance, [39] showed that olfactory stimulation was associated with a stronger feeling of being brought back to the past, but it did not trigger more vivid memories. In our study, subjective reliving was measured on a scale including six aspects of subjective experience: mental time travel, truthfulness of memory, imagery, emotion, self-reference and spatio-temporal context. We therefore conclude that odor exposure had a beneficial effect on the subjective reliving of autobiographical memories in AD. 

From a neuro-anatomical point of view, the beneficial effects of odor on the emotional and phenomenological properties of autobiographical memories may be due to the proximity between the olfactory cortex and the amygdalo-hippocampal complex, which is known to be involved in the consolidation of the memory trace [70]. The amygdala plays an important role in the formation of emotional memories and modulates autobiographical memory [71]. Thus, given the proximity between the olfactory cortex and the limbic structures [28], odor is a preferential candidate for stimulating the modular function of the amygdala. This anatomical proximity may provide a more direct access to the spatiotemporal and phenomenological details of memories compared to other modalities. 

In our study, autobiographical specificity was higher in AD participants after odor exposure than in an odor-free condition. In other words, odor-evoked autobiographical memories are more anchored in a spatiotemporal context than memories retrieved without odor. As suggested by [2], this result can be interpreted within the framework of the encoding specificity of memory hypothesis [72], which considers that “sensory elements of the encoding context are processed along with the target information as part of the memory trace”. Therefore, the presentation of an odor may serve as a useful cue to retrieve the target information. However, as suggested by context-dependent studies [73], this interpretation is valid only if the same odor is presented at encoding and retrieval. We asked our participants to retrieve any memory that came to mind, not necessarily those connected to the olfactory stimulus, so it is difficult to know whether the olfactory stimulus was present at encoding. Another possible explanation was provided by [1], i.e., the presentation of a powerful sensory cue (odor) at retrieval triggers an associative process during autobiographical retrieval. The memory trace might be activated via an automatic process characterized by a fast interaction between the olfactory cue and the memory [1]. Thus, our results suggest that the presentation of an odor could encompass generative and complex remembering by promoting the automatic recovery of autobiographical memories in AD, resulting in more detailed personal events. 

We further investigated the links between the characteristics of odor-evoked autobiographical memory (arousal, valence, subjective experience, and specificity) and depression scores in AD. We found negative correlations between arousal, subjective reliving and emotional valence of odor-evoked autobiographical memories and depression, suggesting that the higher the depression score was, the more autobiographical memories were associated with poor reliving and were rated negatively by AD participants. These findings suggest that depression may be associated with the emotional and phenomenological characteristics of odor-evoked memories in AD. In general, depression is one of the main psychiatric symptoms in AD, and it seems to be intimately linked with the behavioral and cognitive characteristics of the disease [40,41,42]. In our study, depression scores in AD were relatively low and no significant difference was observed between AD and control participants. This may explain why odor exposure triggered a higher number of positive memories in AD patients despite the presence of depressive symptoms. Furthermore, even though we found no significant differences between depression scores in AD and control participants, depression seems to have a greater association with odor-evoked autobiographical memories in AD. This may be due to the different cognitive and emotional mechanisms involved in the recovery of odor-evoked autobiographical memories in AD patients and healthy older adults. 

Interestingly, odor exposure did not improve autobiographical memory in controls, unlike in AD participants. However, we found no significant interaction between group and condition for the arousal, the emotional valence and the subjective reliving, preventing us to conclude to a differential impact of olfactory stimulation in AD and control participants. The only significant interaction between group and condition was observed for specificity, which means that odor stimulation is more efficient to trigger specific autobiographical memories in AD patients than in control participants. The absence of beneficial effect in control participants might be due to a ceiling effect as controls did not experience any difficulty in retrieving specific autobiographical memories, even in the absence of olfactory stimulation. Another explanation is that the scales used to assess autobiographical characteristics were not sensitive enough to detect any difference in controls across the experimental conditions. This would be particularly true for the specificity scale which is sensitive to ceiling effects. In a future study, it would be interesting to replicate these results with more sensitive tools. 

One limitation of our study is that subjective experience and emotional experience were measured using self-report. Future research should compare the characteristics of autobiographical memory using self-report and a more objective method such as voice-recording and physiological responses such as galvanic responses of heart rate variation. It would thus be possible to distinguish between felt (subjective) and lived (objective) emotions or subjective experience. Moreover, although cinnamon was used for the reasons described above [54], olfactory function was not tested in any of the participants. Another potential limitation is that only one smell was used. If several were to be tested, the participants’ choice might be meaningful to them and trigger more detailed autobiographical memories. Finally, our sample is mainly composed of females, several research demonstrated that women generally outperformed men in olfactory abilities (see [74] for review). Future studies should control for sex differences in olfactory abilities. 

## 5. Conclusions

Regardless of these limitations, this study is the first to demonstrate the positive effects of olfactory stimulation on the emotional properties of autobiographical memory, since AD patients reported more positive memories when they were cued by an odor. Future studies should replicate these findings investigating whether the positive odor-evoked autobiographical memories in AD should help patients to maintain a positive self-image in the disease. More importantly, future epidemiological research is needed to understand the complex mechanisms between olfaction, memory and emotion in AD.

## Figures and Tables

**Table 1:** Demographic, neuropsychological and clinical characteristics of Alzeimer’s Disease (AD) and control participants.

		AD (*n* = 25)	Controls (*n* = 23)
Women|men		22|3	18 | 5
Age in years		82.04 (7.34)^n|s^	80.91 (9.87)
Education in years		10.36 (2.55)^n|s^	10.78 (2.63)
Depression	Hospital Anxiety and Depression Scale	3.72 (1.99)^n|s^	2.91 (2.55)
General cognitive functioning	Mini-Mental State Examination	19.32 (3.68) **	27.78 (1.41)
Episodic memory	Grober and Buschke	3.04 (2.71) **	8.56 (2.59)

*Note:* Standard deviations are given between brackets. Performance on Mini-Mental Sate Examination refers to correct responses/ 30. Performance on Grober and Buschke task refers to correct responses/ 16. Maximum score on depression scale was 21 points. ^n|s^ differences between groups were non-significant. Differences between groups were significant at ** *P* < 0.001.

**Table 2:** Number and frequencies of positive, negative and neutral autobiographical memories in AD and control participants in the odor and the odor-free conditions.

	AD Group				Control Group			
	Odor-Free		Odor		Odor-Free		Odor	
	*N*	%	*N*	%	*N*	%	*N*	%
Emotional valence								
Positive	12	48	19	76	21	91.3	22	95.6
Negative	9	36	2	8	1	4.35	1	4.35
Neutral	4	16	4	16	1	4.35	0	0
